# A Machine Learning Approach for the Detection and Characterization of Illicit Drug Dealers on Instagram: Model Evaluation Study

**DOI:** 10.2196/13803

**Published:** 2019-06-15

**Authors:** Jiawei Li, Qing Xu, Neal Shah, Tim K Mackey

**Affiliations:** 1 Department of Healthcare Research and Policy University of California - San Diego, Extension La Jolla, CA United States; 2 Global Health Policy Institute La Jolla, CA United States; 3 Department of Anesthesiology University of California - San Diego, School of Medicine La Jolla, CA United States; 4 Division of Infectious Disease and Global Public Health University of California - San Diego, School of Medicine La Jolla, CA United States

**Keywords:** opioids, social media, narcotics, substance abuse, machine learning, internet, prescription drug abuse, artificial intelligence

## Abstract

**Background:**

Social media use is now ubiquitous, but the growth in social media communications has also made it a convenient digital platform for drug dealers selling controlled substances, opioids, and other illicit drugs. Previous studies and news investigations have reported the use of popular social media platforms as conduits for opioid sales. This study uses deep learning to detect illicit drug dealing on the image and video sharing platform Instagram.

**Objective:**

The aim of this study was to develop and evaluate a machine learning approach to detect Instagram posts related to illegal internet drug dealing.

**Methods:**

In this paper, we describe an approach to detect drug dealers by using a deep learning model on Instagram. We collected Instagram posts using a Web scraper between July 2018 and October 2018 and then compared our deep learning model against 3 different machine learning models (eg, random forest, decision tree, and support vector machine) to assess the performance and accuracy of the model. For our deep learning model, we used the long short-term memory unit in the recurrent neural network to learn the pattern of the text of drug dealing posts. We also manually annotated all posts collected to evaluate our model performance and to characterize drug selling conversations.

**Results:**

From the 12,857 posts we collected, we detected 1228 drug dealer posts comprising 267 unique users. We used cross-validation to evaluate the 4 models, with our deep learning model reaching 95% on F1 score and performing better than the other 3 models. We also found that by removing the hashtags in the text, the model had better performance. Detected posts contained hashtags related to several drugs, including the controlled substance Xanax (1078/1228, 87.78%), oxycodone/OxyContin (321/1228, 26.14%), and illicit drugs lysergic acid diethylamide (213/1228, 17.34%) and 3,4-methylenedioxy-methamphetamine (94/1228, 7.65%). We also observed the use of communication applications for suspected drug trading through user comments.

**Conclusions:**

Our approach using a combination of Web scraping and deep learning was able to detect illegal online drug sellers on Instagram, with high accuracy. Despite increased scrutiny by regulators and policymakers, the Instagram platform continues to host posts from drug dealers, in violation of federal law. Further action needs to be taken to ensure the safety of social media communities and help put an end to this illicit digital channel of sourcing.

## Introduction

### Background

In June 2018, the US Food and Drug Administration (FDA) held the *Online Opioid Summit*, a 1-day meeting seeking to generate momentum around the need to combat illicit internet sales of opioids [1]. In addition to federal agencies, several internet and social media companies were in attendance, including Google (which operates YouTube and Google+), Twitter, Facebook (which operates Instagram and WhatsApp), Pinterest, and other e-commerce, technology, and patient safety organizations [2]. The meeting was organized to facilitate cooperation among these stakeholders to address illicit internet opioid sourcing and diversion, a challenge that adds fuel to a national public health emergency that claims the lives of an average of 130 people daily from addiction-related overdose [3].

Importantly, federal law explicitly prohibits the internet sale of controlled substances as enforced by the 2008 Ryan Haight Online Pharmacy Consumer Protection Act (RHA) [4,5]. Named after a Californian adolescent who died in 2001 after overdosing on Vicodin purchased from an online drug seller without a prescription, the RHA was meant to curb the use of the internet as an alternative and convenient channel of sourcing [6]. However, since Mr Haight’s death, the internet ecosystem has rapidly proliferated and diversified, now populated by illegal internet pharmacies, social media posts from illegal sellers, and dark Web vendors, all who have been implicated in illegal online opioid sales [6-12].

Though illegal prescription drug sales are often found by users through search engine results and internet pharmacy advertisements (including spam email), popular social media platforms have emerged as a direct-to-consumer marketing tool for illegal sellers [2,12-15]. Previous research and investigative reporting have detected illegal opioid sales and drug dealing on several social media platforms, including Twitter, Facebook, and Instagram [7,12,16-21]. As Twitter provides a convenient way of accessing data through its application programming interface (API), many substance abuse infoveillance studies have focused on this microblogging platform [22-25]. Our own prior studies used both supervised machine learning classifiers and an unsupervised topic model to detect internet pharmacies selling opioids (including fentanyl) [6,7,21,26]. Others have primarily focused on analyzing Twitter messages for opioid and substance abuse behavior with manually annotated data, examining social circles of users, measuring user sentiment, using natural language processing, and using deep learning [22,23,27,28]. Other studies have used deep learning models to detect and describe adverse drug reactions via Twitter [29,30].

However, there are far fewer studies that have conducted infoveillance research on the Instagram platform, likely because of the difficulty of collecting Instagram data and the different data features that require additional data cleaning and processing. Instagram is an image and video sharing social media platform (reaching 1 billion monthly users in 2018) and is particularly popular among young adults (ie, 71% of those aged between 18 and 24 years), a critical demographic for substance use initiation [17]. Prior studies by Zhou et al have analyzed Instagram data primarily for substance abuse behavior and did not use deep learning models but instead used other machine learning approaches [28]. One relevant study by Yang and Luo used a model based on multitask learning that analyzed both images and text to track and classify drug abuse–related posts, including differentiating for drug dealers [17]. The study analyzed the user timelines of identified posts to differentiate drug dealers from users who exhibited drug use behavior and achieved a high classification accuracy of 88% [17].

### Objective

Building on these prior studies that have used different big data and machine learning approaches to detect substance abuse behavior and illegal drug selling on social media, this study describes the use and evaluation of a deep learning model to better automate the detection of illegal opioid and other illicit drug sales on Instagram. Our study focuses on detecting illegal drug selling posts (not accounts) from hashtag searches and using deep learning to analyze text from posts.

## Methods

### Overview

**Figure 1:**
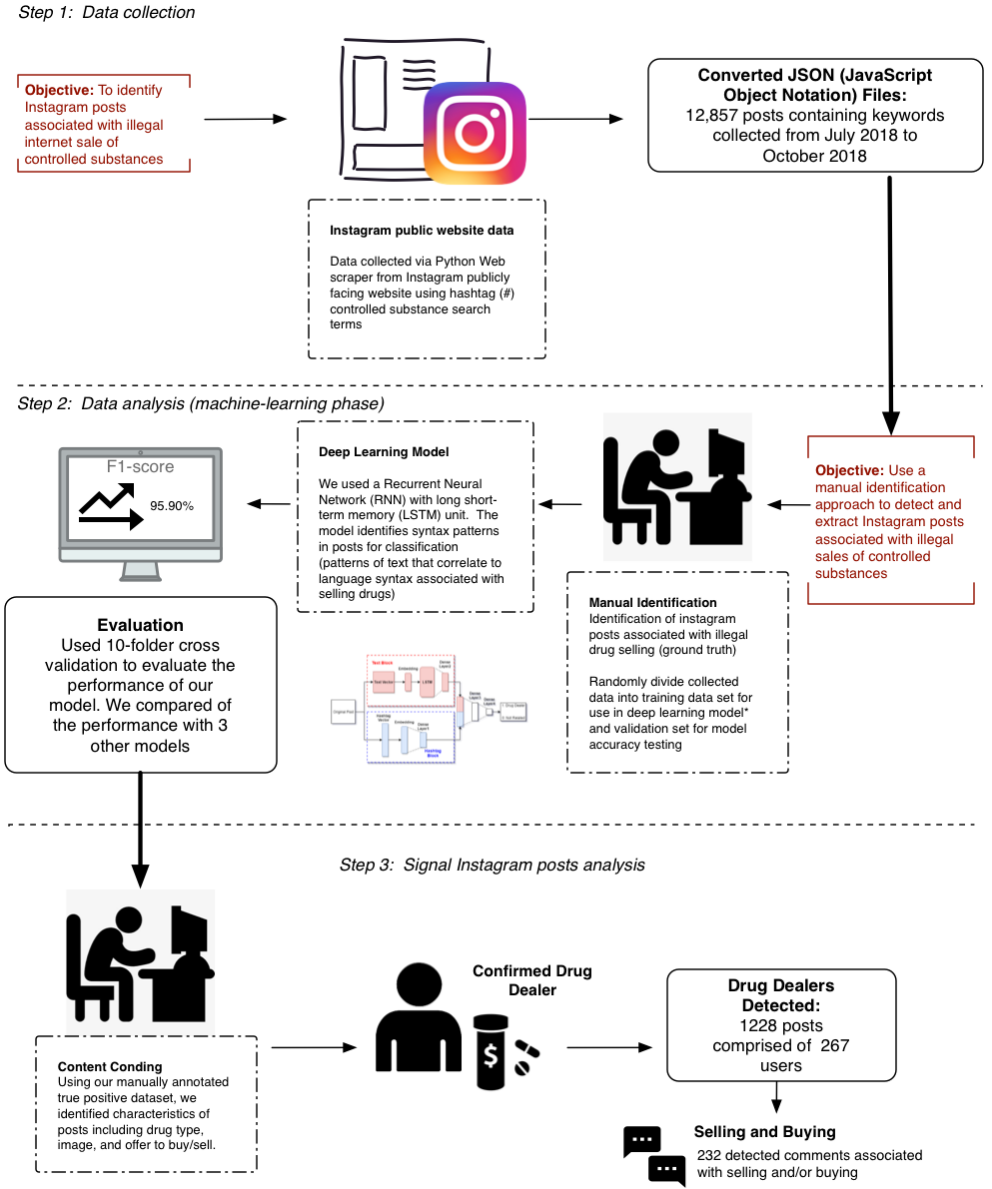
Summary of study methodology.

Our study comprised 3 phases: data collection, data processing, and model training. The goal of our study was to develop and evaluate a machine learning approach that has the best performance for identifying illicit opioid drug selling via Instagram (Facebook, Inc.). To accomplish this, we first collected a set of posts that contained suspicious drug selling behavior by conducting automated searches for opioid-related hashtags and posts. We then used posts detected in these hashtag searches as a training set for our deep learning model, so we could better enable detection of posts in the entire corpus of all messages collected (see Figure 1 for summary of methods). Importantly, Instagram is a platform that has different ways of presenting messages from its users. For other social media platforms such as Twitter and Facebook, the main content in the post is often the text accompanied by hashtags. However, on the basis of the method of searching for messages on Instagram (ie, users search for posts and topics by hashtags), the more hashtags a post contains, the easier it will be found. Therefore, a post for Instagram usually comprises 3 main pieces of content: text, hashtags, and an image. In this study, we will analyze the performance of models examining text and hashtags to determine what is the best approach to detecting illegal drug dealers.

### Data Collection Phase

Since July 2018, Instagram has disabled many functions of its public API and has set limitations on data collection. To collect a more representative dataset than what is available from its limited public API, we developed a method to Web scrape results from the Instagram platform website based on opioid keyword hashtags (a word or phrase preceded by a hash character, #) search results. Our Web scraper was built in the computer programming language Python and converted source code from the Instagram website into JavaScript Object Notation (JSON) data files. Data scraped from the website included the text of the Instagram post, comments to the post by Instagram users, and associated metadata (eg, date, time, and user profile information).

As search on Instagram is conducted using hashtags (eg, #subject), we chose an initial list of hashtags related to controlled substances identified via manual searches and then populated these opioid-related hashtags into our automated search and Web scraping data collection process. This allowed us to discover a greater number of opioid and drug-related hashtags that are present in the Instagram community. Our initial set of hashtag keywords included *xanaxangel*, *percocert* (Percocet misspelled), *adderrall* (Adderall misspelled), and *hydrocodoneacetaminophen* (misspelled.) These keywords relate to hashtags associated with common controlled substance drug names that were used on Instagram at the time of the study. Many of these keywords are misspelled as Facebook and Instagram currently disables search results for certain explicit opioid keywords in search queries [2]. However, alternative opioid hashtag keywords are relatively easy to find, including some that are derived from the platform’s own suggested alternative hashtags when conducting searches [31].

Importantly, hashtags used by drug dealers are different depending on what type of drug(s) they are selling. Hence, it is important to expand the number and diversity of possible drug-related hashtags to collect a better sample of data to analyze. To increase the number of hashtags likely related to drug dealing, we examined search results for our initial set of hashtags using a 2-loop process during our automated search and Web scraping. In the first loop, we captured data from an individual post under a certain hashtag in the initial set of hashtags used. Our Web scraper continued to collect the JSON data from the source code for each of these posts until it reached a set limitation (in this study we ended our search loop when the post was older than 3 months). We then filtered out the hashtags from each post and chose other hashtags that contained keywords associated with controlled substances and illicit drugs by manually inspecting all hashtags collected in the loop. Hashtags that contained opioid and drug-related keywords or combinations thereof were then added to the hashtag list so they could be searched in a second loop that would go through all the hashtags identified. A full list of hashtags identified in this study is available in Multimedia Appendix 1.

Data collection occurred from July to October 2018, with hashtags limited to English language and with no set geographic or other filters for posts. In our data cleaning process, we also discarded duplicate posts that were replicated when collecting posts under different hashtags search loops, and then removed hashtags and stop words before textual analysis in the data processing phase described below.

### Data Processing Phase

An Instagram post generally contains content with the user’s text (ie, message) and also hashtags to self-curate the content and associate it with other user posts and comments. Unlike normal text, hashtags can be placed randomly in the post, which could affect the accuracy of the machine learning model used in this study, as our deep learning model is based on learning the pattern of the text. To address this, we took the following additional steps to process our data:

1. We eliminated duplicate results of posts that appeared in multiple hashtag searches.
2. From each post, we extracted the text and removed any hyperlinks, special characters (eg, emoji, !, @), and stop words (eg, hers, between, yourself). We used the Natural Language Toolkit package in Python to remove stop words. We did not exclude # in this study, as # represents the hashtags of keywords that we specifically wanted to analyze.
3. We then built a dictionary based on the words in all texts; each word had a corresponding index. Then we transformed the word into an index in each text.
4. We kept text that had more than 1 index after step 2 as original data; then we removed all the hashtags from the original data and removed the texts that had no remaining words left. These data are referred to as no hashtag data.
5. We eliminated the duplicate texts in each dataset after step 3. Hence, all the texts remaining were unique with a different pattern.
6. We then manually annotated all the posts detected (including text and images) to identify and classify posts that involved illegal drug dealing or selling to establish ground truth for model evaluation. We accomplished this by using a binary coding scheme of yes or no based on assessing whether a post contained text or image information about a prescription opioid, controlled substance, or other suspected illicit drug product and that the post also included contact information on how to trade or purchase the drug from the dealer. The second and the third author coded posts independently and achieved a high intercoder reliability for results (kappa=0.98). For inconsistent results, both authors met and reviewed the posts together with the last author (a subject matter expert in internet substance abuse behavior) and all authors conferred on the correct classification of the post.
7. Using the manually annotated ground truth data in step 6, we then evaluated the performance of a deep learning model to identify illegal drug sellers by comparing it with other machine learning models.

### Model Training

In this study, we used 4 supervised models to analyze our Instagram data: decision tree, random forest (RF), support vector machine (SVM), and a deep learning model we developed [32-34]. The first 3 models are traditional machine learning models, which perform well for classification tasks [35-37]. For this study’s deep learning model, we used a recurrent neural network with long short-term memory (LSTM) unit to study the pattern of text in a post. This model is well-suited for classifying, processing, and making predictions based on time series data and also exhibits high performance on speech text analysis [38-41].

**Figure 2:**
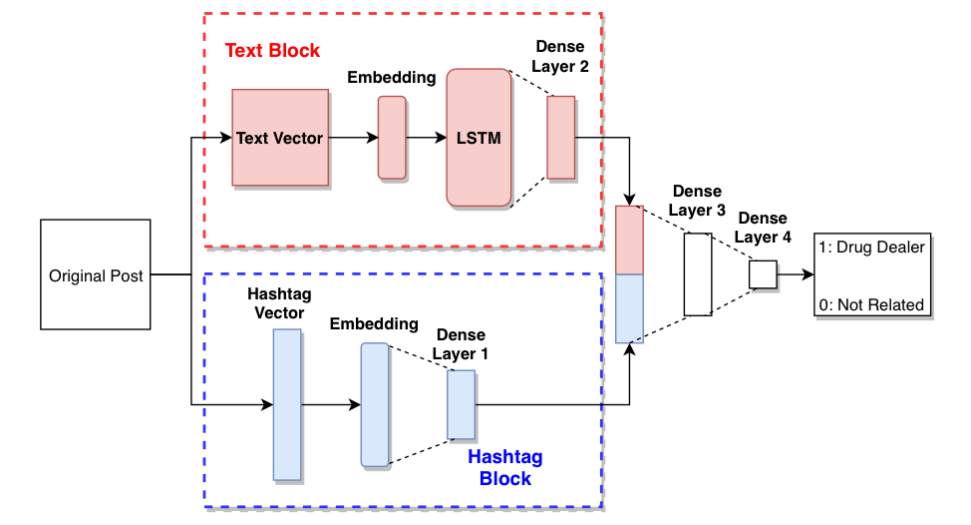
Structure of deep learning model. Embedding layer: input_dim is 29,832, which is the size of the dictionary; the input_length for text is 50, input_length for hashtag is 15; the output_dim is 400. Long short-term memory layer: contains 800 units, with dropout=0.2, recurrent_dropout=0.2. Dense layer 1: unit=200, activation=sigmoid. Dense layer 2: unit=200, activation=sigmoid. Dense layer 3: unit=200, activation=sigmoid. Dense layer 4: unit=1, activation=sigmoid. Optimizer: Adam (learning rate set at 0.0001). Loss: Binary_crossentropy. LSTM: long short-term memory.

The structure of the model is shown in Figure 2. It contains 2 major parts, text block and hashtag block. The text block was used to store the information in the text only, the hashtag block was used to store the information from the hashtags only. When we input the data, we separated the original text into the text part and hashtags part, and for each part we generated a vector based on the dictionary we built in the data processing stage. The length of the text vector was 50 words, which is the average length of texts in our dataset (if the size of the vector is too large, it will increase the workload for the model; if it is too short, we may lose signal data). If the text was longer than 50 words, we only chose the first 50 words to maintain the efficiency of the model; if it was shorter, we filled the rest of the vector with 0. Then we used word embedding to generate the text into a word vector with dimensions (50, 400). The strategy for hashtags was the same, but the average length of hashtags was set to 15 characters.

The text block contains 1 layer of LSTM combined with 1 fully connected network (FCN) [42]. LSTM is a recurrent neural network that can be used to predict the next word based on the current word. Hence, it will learn the correlation between different words, which is the pattern of the text, during the training process. The FCN (dense layer) is used to learn features from the original data and to keep as much information as possible in another vector (in our study we kept the output from the former layer into a smaller dimension vector). The dense layers 1 and 2 in these 2 blocks will keep the signal from text to a much smaller vector. We then merged these 2 vectors together and used another 2 FCNs (dense layers 3 and 4) to generate the prediction.

Every time the model gives a prediction (possibility for 1=drug dealer and 0=not related; if the possibility is larger than 0.5, we consider it as drug dealer), the model will calculate the difference between the prediction and the label; the difference is referred to as the loss. If the loss is low, that means the model is performing well on prediction. There are many ways to calculate the loss; in our model, we used binary cross-entropy loss [43]:

Loss=−(y×log(p)+(1−y)×log(1−p))

This is the format for a binary class only; y represents the ground truth of each text (either 0=not drug dealer or 1=drug dealer), p represents the predicted probability that the text is of the class. The loss shows the difference between our prediction and ground truth.

After we get the loss, the model will run back propagation to update the parameters in LSTM and FCN to decrease the loss value [44]. The updating is based on the learning rate and optimizer we choose. The learning rate is used to keep the learning process in an acceptable range. If the learning rate is too small, it will take a longer time for the model to achieve the best parameters; if it is too large, it may never reach the local minimum for the loss. Therefore, an Adam optimizer algorithm was used for back propagation, which allows the learning rate to adapt based on the parameters which will make large updates for infrequent parameters and small updates for frequent parameters [45].

During the updating process, the model will learn how to distinguish what a drug dealer’s post looks like. On the basis of this pattern, the model can identify syntax patterns in posts to classify them as signal posts (patterns of text that correlate to language syntax associated with selling drugs) and can then separate posts that contain relevant hashtag keywords, whose syntax is not related to drug selling.

The data were separated into a training set and validation set; the training set was used to update the model and the validation set was used to evaluate the performance of our model, which we discuss further in the Model Evaluation section. In the training process, the deep learning model will keep looping on learning the training set to reach the minimum of the loss. However, this process could cause overfit, which will make the model too sensitive to the pattern of the texts in the training set. To prevent overfit, we need another dataset that is different from the training set (ie, the validation set). If the loss of the validation set is increasing, it means that the model learned too much from the training set and the training needs to be stopped. In our model, we compare the most recent validation loss with all 4 previous validation losses; if it is larger (the loss is continually increasing), the model will stop training.

### Ethics Approval and Consent to Participate

Ethics approval and consent to participate were not required for this study as data derived for this study were available from public sources and there were no interactions with social media users.

### Data Availability

The pretrained model for this study is available on GitHub under the user account name Mathison under the file Instagram_drugdealer_detection.

## Results

### Model Evaluation

We collected a total of 12,857 Instagram posts over a 3-month period (July 19, 2018 to October 18, 2018) that we used for analysis. There were a total of 1228 drug dealer posts based on the manual annotation we conducted, which comprised 267 unique users. As of October 18, 2018, 206 of these posts were still active and viewable on the Instagram platform. As the volume of the target posts is low compared with the total dataset, we used 10-folder cross-validation to evaluate the performance of our model. This method is used to estimate how the model is expected to perform when facing limited samples. We shuffled each dataset (original data and no hashtag data) randomly and separated them into 10 folders—9 of them are the training set (in the deep learning model we used 70% as the training set and 30% as the validation set) and the rest are the test set used to assess the area under the curve (AUC), precision, recall, and the F1 score of the model. When calculating the AUC, we used the whole dataset rather than the cross-validation. There were a total of 10 iterations; for each iteration, we chose a different folder as the test set. Hence, this allowed us to ensure that each text could be used to make prediction. For each iteration, the model was reset. After we finished all iterations, we could calculate the average score for each model.

For the deep learning model, to prevent the problem of overfit, we separated the original training set into 2 parts, 70% of the set as the training set and 30% as the validation set. This allowed us to ensure that the model we generate from each iteration can have the best performance on the test set.

The results from our model evaluation are separated into 3 parts according to the data we preprocessed (Table 1): text with hashtags, hashtags only, and text without hashtags.

On the basis of this evaluation, the deep learning model has the best performance compared with the other 3 models based on the F1 score. However, the precision of the deep learning model does not show a better result than RF or SVM, suggesting that deep learning is not more effective at filtering for false positives.

**Table 1:** Performance for each model based on variations of text and hashtag use.

Performance measure		Decision tree	Random forest	Support vector machine	Study model
**Text with hashtags, %**					
	Precision	95.05	96.00	96.86	94.81
	Recall	82.15	86.08	81.21	91.42
	F1 score	88.13	90.77	88.35	93.09
	Area under the curve	96.67	96.85	97.18	98.12
**Hashtags only, %**					
	Precision	86.22	94.14	95.39	89.60
	Recall	86.50	87.13	84.24	88.89
	F1 score	86.36	90.50	89.47	89.24
	Area under the curve	95.95	95.23	95.43	94.32
**Text without hashtags, %**					
	Precision	88.49	97.07	97.80	93.60
	Recall	93.08	91.31	89.32	98.31
	F1 score	90.73	94.11	93.37	95.90
	Area under the curve	95.56	94.85	93.49	99.12

The possible reason is that drug dealers on Instagram usually have similar distinct text formats of selling drugs, which includes providing contact information with a phone number or email address. Therefore, once the pattern of the text is established, the deep learning model can identify these posts with greater ease. This is also shown in the results; the recall of the deep learning model is much higher than in other models. However, the text patterns of other nonrelated posts are not similar, so the deep learning model may be confused by random patterns which makes its precision, compared with other models, lower.

Table 1 shows that the model performance on hashtags only does not improve when compared with performance on texts with hashtags, especially for the deep learning model. However, when we use the text without the hashtags, all the scores increased except for the recall of the deep learning model compared with performance on texts with hashtags (Table 1). These performance results suggest that removing hashtags can increase the accuracy of the model, but may increase false positives for the deep learning model. This may occur when the user is engaging in nondrug related sales, but uses the same text pattern with different hashtags than drug dealers, as removal of hashtags makes these texts patterns appear more similar to posts from actual drug dealers.

### Text Analysis of Drug Dealer Posts

When manually annotating the text contained in the 1228 drug selling posts, we identified 2 forms of communication by users: (1) the use of hashtags (#) in front of illicit drug-related keywords to self-curate content and (2) descriptive language of drug selling–related activity in combination with related images posted by users. Each post can contain a combination of types of text (eg, hashtags and/or descriptive text), images or videos, and other metadata associated with the Instagram user. The vast majority of posts (1196/1228, 97.39%) had hashtags, whereas 32 out of 1228 posts (2.61%) only had descriptive language with no hashtags, which we suspect were posts modified after data collection occurred and before manual annotation.

The majority of hashtags detected in signal posts were related to the controlled substance Xanax (Alprazolam, a nonopioid controlled substance; 1078/1228 posts, 87.78%), followed by oxycodone/OxyContin (321/1228, 26.14%) and illicit drugs lysergic acid diethylamide (LSD; 213/1228, 17.34%) and 3,4-methylenedioxymethamphetamine (MDMA; 94/1228, 7.65%; see Table 2 for summary).

**Table 2:** Number of posts related to controlled substance hashtags (N=1228).

Drug name^a^	Hashtag^b^	Posts, n (%)^c^
Xanax	#xanax	802 (65.3)
	#xanaxfamily	530 (43.1)
	#2mgxanax	321 (26.1)
	#zanax	112 (9.1)
	#greenxanax	84 (6.8)
	Total	1078 (87.8)
Oxycodone/OxyContin	#oxycodone	30 (2.4)
	#oxycodine	261 (21.3)
	#oxy80s	213 (17.3)
	#oxycontin	215 (17.5)
	#oxicotin	233 (18.9)
	#oxicodone	212 (17.2)
	Total	321 (26.1)
Lysergic acid diethylamide	#LSD25	138 (11.2)
	#LSDtabs	130 (10.5)
	Total	213 (17.3)
3,4-Methylenedioxy-methamphetamine	#mdmapills	50 (4.1)
	#mdmaforsale	21 (1.7)
	#mdmazing	40 (3.3)
	#mdmaonline	21 (1.7)
	Total	94 (7.6)

^a^Drug name column relates to drug detected in the image and text of the post.

^b^Hashtag refers to the presence of a hashtag in a post detected.

^c^Posts is the number of posts with the hashtag and the percentage of total posts that contained the hashtag.

When manually annotating posts that contained hashtags, we found that the use of hashtags can be categorized into 3 major groups: (1) hashtags with controlled substance drug names and other illicit drug names, (2) slang or other codewords used in the Instagram user community for specific controlled substances and substance use behavior, and (3) other keywords and codewords describing selling and other promotional behavior (eg, shipping and selling). Examples for each of these categories are provided below:

- Drug name: Generic or brand name of a drug in a hashtag (eg, #xanax, #oxycodone, #oxycontin, #LSD, #MDMA)
- Codewords: Codewords for controlled substances, including (1) misspelled drug name, for example, “#zanax”, (2) extended drug name, for example, “#mdmaforsale”, “#2mgxanax”, and (3) street name of drug, for example, “#whitebar”
- Keywords related to sale or shipping, for example, “#forsale”, “#shipping”, etc

When assessing posts with descriptive text describing actions or behaviors of drug dealers, we were able to classify posts into 2 additional categories: (1) drug sale promoting language, for example, “interested in placing order without prescription”, “order now for quick delivery” and (2) contact information of purported drug seller. On the basis of these 2 categories, Instagram drug dealers appear to clearly indicate in their descriptive text of their posts an offer for sale and also provide other users with information on how to contact the seller. Contact information generally included an email address, phone number, or user account information about a communication-based application or mobile app. Among posts containing communication applications or apps, Wickr (445/1228, 36.23%), Telegram (245/1228, 19.95%), Kik app (225/1228, 18.32%), and WhatsApp (188/1228, 15.30%) were utilized. In some cases, a drug seller might also include descriptive text in the post referring to their account profile to reference contact information instead of providing it in the text of the post.

From the 1228 detected posts we analyzed, 232 of these posts explicitly included an offer for sale and offer to buy by the users. In this case, there was an Instagram post or comments within a post from a user offering to sell drug(s) (with contact information) and a comment from another user that asked for more information or offered to buy the drug.

### Manual Image Annotation of Drug Dealer Posts

In addition to text analysis, we also collected a total of 260 pictures from the 1228 posts analyzed. These images were manually annotated to determine if they were related to controlled substances or illicit drugs (primarily coded for whether they included a picture of a controlled substance or suspected illicit drug product) or if they were unrelated images. A total of 252 images (252/260, 96.9%) included pictures or images of different types of drugs that can be categorized into 5 different categories.

**Figure 3:**
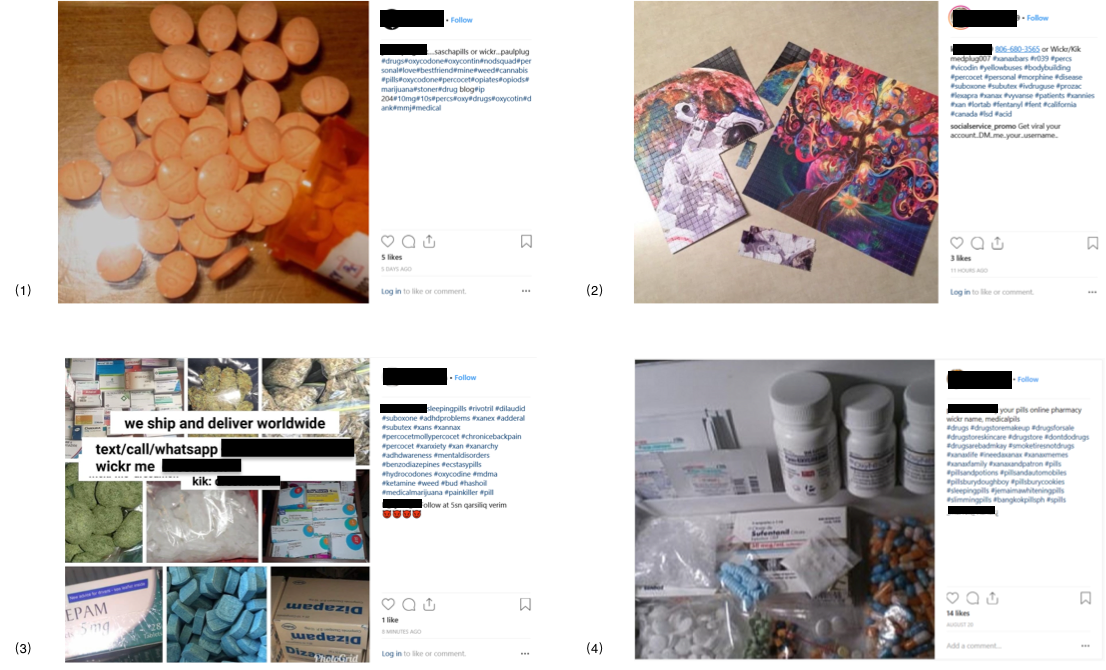
Examples of Instagram posts of illegal drug sale categories (user information and text from post have removed). (1) A post of prescription drugs; (2) a post of lysergic acid diethylamide (LSD); (3) a post with written contact information imbedded in the image; and (4) post with multiple drug types.

In the first category which included prescription-controlled substances only, there were 175 posts (175/260, 67.3%) comprised of the following controlled substances: Xanax (41 posts), Alprazolam (34 posts), oxycodone/OxyContin (25 posts), Adderall (17 posts), and amphetamine and dextroamphetamine (14 posts). In the second category that contained images of illicit drugs only, there were 60 images comprising LSD (19 posts, including blotter paper soaked in LSD), ecstasy/MDMA (14 posts), cannabis (13 posts), and magic mushrooms (7 posts; see Figure 3).

**Figure 4:**
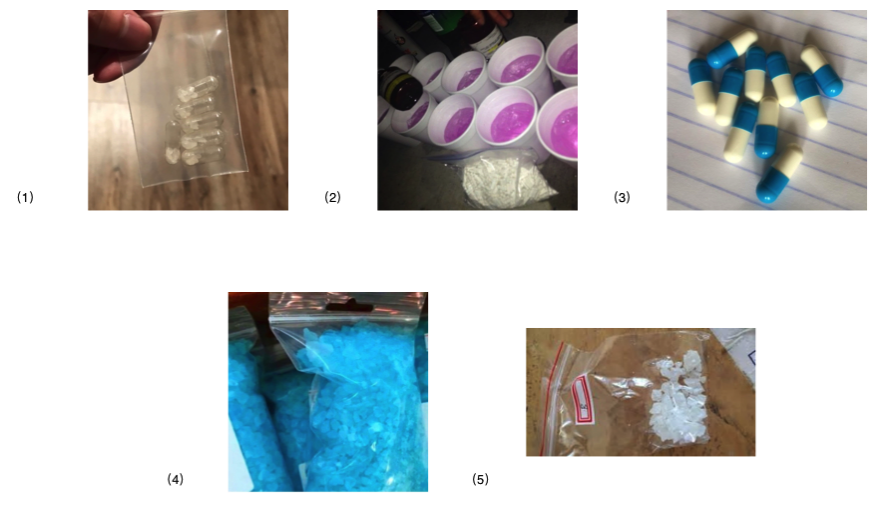
Examples of Instagram posts with suspected drug products (user information removed). The 5 unclassified pictures include (1) clear capsules with white crystalline granules, (2) cups with pink liquid, (3) blue and white capsules with no drug identification, (4) plastic bags of blue crystals, and (5) a bag of white crystals with a label “B”.

In the third category, there were 15 images that contained both a prescription drug and illicit drug. In the fourth category, there were 5 images we could not classify, but which we suspected as either illicit drugs or other drug-related manufacturing materials (see Figure 4.)

**Figure 5:**
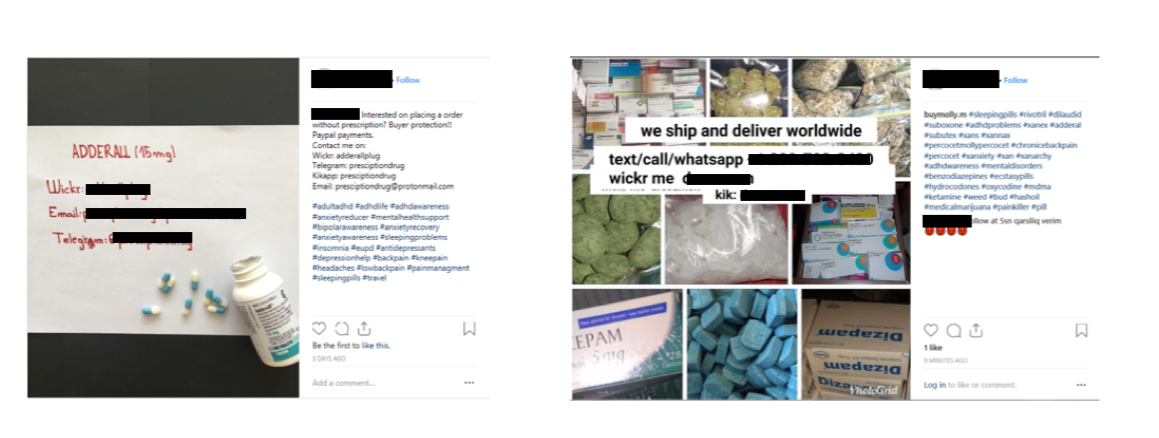
Examples of Instagram posts with written contact information. There were 60 images that included either typed or written contact information.

Finally, in the fifth category, separate from images of drug products, we also detected images that included typed or written information on a physical medium communicating the drug dealers’ contact information (see Figure 5).

## Discussion

### Principal Findings

Over a 3-month period, we used a combination of hashtag keyword searches, Web scraping, deep learning, and manual annotation to detect and characterize 1228 posts from 267 unique users that we suspected were associated with illegal drug dealing on Instagram. Several prescription-controlled substances, illicit drugs, and other suspected drug products were detected as being offered for sale purportedly by drug sellers/dealers. In addition, we observed Instagram users having conversations via comments purporting to both offer a sale of a drug product and receiving an offer to buy from another user (ie, generally a comment from a seller to another user to contact them directly via a third-party communication application to enter into a drug-related transaction.)

These initial results are alarming and generally conform to existing nonscientific investigational news reports on the subject, in addition to published research on illegal drug sales on this and other social media platforms such as Twitter [6,7,17]. As a result of emerging evidence linking the risks of illicit drug access and diversion via social media technology there has been increased public attention and scrutiny. Congressional bodies, including the House Energy and Commerce Committee, have called on social media company executives to explain why their platforms facilitate this illegal activity [46,47]. This includes sharp inquiry from Congressman David McKinley (West Virginia), whose state has been heavily impacted by the opioid epidemic. Mr McKinley questioned both Twitter co-founder and Chief Executive Officer Jack Dorsey and Facebook Chairman and Chief Executive Officer Mark Zuckerberg in 2018 congressional testimony on what steps their platforms were taking to remove illegal opioid sellers and how they will protect the public [47,48]. Mr Zuckerberg responded that the enormity of data makes it hard to monitor content and that *artificial intelligence* approaches to *proactively* find content were needed [47].

In April 2018, Facebook and Instagram took action by blocking opioid-related hashtag searches on their platform and reportedly suspending accounts [16,20]. However, our study indicates that drug sellers continue to populate Instagram despite these actions and that these communities have changed their use of hashtags possibly to avoid detection. Hence, to carry out the legislative intent of the RHA to promote patient safety and prevent substance abuse behavior, there is clear need for innovative technology solutions that have high accuracy and are scalable and can help all parties (including technology companies, regulators, and law enforcement) detect, classify, and take action against digital drug dealers.

### Study Limitations

Our study has certain limitations. First, this study was limited to a short period of data collection and we did not purposely sample Instagram accounts or consider user characteristics such as age, gender, or other demographics. Hence, study results are not generalizable and are not necessarily representative of the Instagram user community. In addition, demographic data are not always readily available in the metadata or the user account information or the post, and if available, may not be accurate. Future studies should assess which specific user communities may be at higher risk for illegal drug sourcing online. We also did not engage with users or verify if drugs purportedly being sold were actually available or sold to other users. Given this limitation, we cannot say with certainty that these drugs were actually being sold. Conducting test purchases of controlled substances and other illicit drugs is prohibited by federal law. However, drug dealers often post pictures of drug products to demonstrate to users that they have availability and we did not observe comments reporting scams or failed drug buys. We also did not use multimodal or synchronous approaches to develop a classification model based on both text and images as used by Yang and Luo, an approach that could improve performance of the model and should be explored in future studies. In addition, though data were collected and analyzed within 3 months of collection, we relied on manual annotation to establish validity of results and evaluate the performance of our model. This time lag because of manual annotation may have resulted in some posts being removed or modified before analysis or manual annotation. Specifically, drug dealers may self-delete posts after they have completed a transaction. Future studies should continue the iterative process of establishing training datasets to inform machine learning models that can more quickly and accurately detect illicit drug dealing.

### Conclusions

In this study, we evaluated a deep learning model to detect drug dealers on Instgram. The deep learning model based on LSTM performed the best compared with the other 3 models evaluated. Furthermore, we compared the deep learning model’s performance with hashtags against messages with only text in Instagram posts and demonstrated that the model yields better results from text without the hashtags, despite the risk of including false positives.

The results of our study further indicate that despite increased scrutiny by regulators and policymakers, the popular social media platform Instagram continues to act as a conduit for opioid, controlled substance, and illicit drug access, a direct violation of the RHA. Importantly, users have active conversations about selling and buying drugs, meaning that these social media posts act as digital marketplaces for drug dealing. Further action is needed to protect the public but needs to be carried out through meaningful collaboration and coordination involving partnership between technology companies, researchers, regulators, law enforcement, and impacted user communities to ensure that the opioid epidemic is not exacerbated by the digital risk environment.

## Appendix

Multimedia Appendix 1 List of hashtag terms used.

## Abbreviations

API: application programming interface
AUC: area under the curve
FCN: fully connected network
FDA: Food and Drug Administration
JSON: JavaScript Object Notation
LSD: lysergic acid diethylamide
LSTM: long short-term memory
MDMA: 3,4-methylenedioxy-methamphetamine
RF: random forest
RHA: Ryan Haight Online Pharmacy Consumer Protection Act, 2008
SVM: support vector machine
