# Supplementary material for: A Machine Learning Approach for the Detection and Characterization of Illicit Drug Dealers on Instagram: Model Evaluation Study
Source: J Med Internet Res. 2019 Jun 15;21(6):e13803. doi: 10.2196/13803 (PMC6598421; doi:10.2196/13803)
Supplement: Multimedia Appendix 1 [file jmir_v21i6e13803_app1.pdf]

Multimedia Appendix

List of hashtag terms used:

#hydrocodoneparacetamol #xanaxparade #xanaxing #xanaxme #xanaxplugdontplae
#thankyouxanax #xanaxitaly #cheloxanaxsiacontuttivoi #liquidxanax.
#xanaxfanclub #Bluexanax #laysxanax #xanaxtape #needaxanax #xanax4sale
#xanaxbarsanddope #xanaxiety #stopdoingxanax2019 #xanaxporn #godblessxanax
#thankGodforxanax #xanaxrap #6xanax #xanaxtakemeaway #xanaxalovesong
#ukxanax #xanaxbars #realxanax #lilxanaxi #xanaxspain #xanaxxr #endxanaxuse
#xanaxflia #xanaxiloveyou #xanaxforgaysummerweddings #happyxanax
#xanaxrepresent #xanaxmania #morexanax #Thexanax #stopdoingxanax2017.
#planetxanax #ineverhadxanaxsoimassuming #stopdoingxanax2018/19 #kawaixanax
#noxanax2019 #xanaxseries #whitexanax. #donttakexanax
#poracciartforbarbiexanax #xanaxplz #thanksxanax #nobutreallyineedxanax
#xanaxpendant #xanaxnap #xanaxtime #xanaxfamily! #xanaxbristol #Xxxanax
#exxanaxcanvas #piùxanaxpertutti #dogxanaxplease #ineedxanax,
#liquidxanaxrecipe #stopxanax2018 #noxanax2018 #xanaxmanaxanax #xanaxgirls
#ineedxanaxandahug #mgxanax #xanaxhawaii #fuckxanax2019 #hatexanax #xanaxgay
#xanaxmeme #xanaxchristmas #barbiexanax #catxanax #Fuckxanax2017
#jakkawaixanax #Fuckxanax #startdoingxanax2017 #adderrall
#xanaxchristmastree #xanaxbars2mgplease #stopxanax2019 #don'tdoxanax
#lifeofxanax #thatisNOTxanax #jualxanax1mg #xanaxaddiction #xanaxturn7
#roxanax #xanaxlove #xanaxrussia #xanax2mg #mensxanaxincorporexanax
#xanaxmovement #xanaxtoday #xanaxtab #xanaxisbadforyou #xanaxteam
#diditakemyxanax #Fuckxanax2018 #ripalldiewithxanax #xanaxArt #spanishxanax
#xanaxalternative #xanaxbarsfoo #takeaxanax #xanaxPlz #CBD>xanax #longxanax
#xanaxfam #rarexanax #mommiesforxanax #xanaxmiamor #xanaxbabygirl #onxanax
#xanaxawareness #betterthanxanax #tomandoxanax #coffeeandxanax #xanaxfamily,
#stopxanaxabuse #simsponxanax #xanaxmanadderall®❤️ #xanaxsdontmakeyou
#xanaxlélek #xanaxFamily #doterraliquidxanax #xanaxchallenge
#rambilovesxanax #xanaxjakarta #xanaxbeat #bluesxanax #dontdropxanax
#antixanaxmovementpioneer #xanaxsoup #saynotoxanaxaftermidnight #xanaxanyone
#xanaxgods #xanaxroller #teamfuckxanax #Stopxanax #mexicanxanax #xanaxlatte
#lilpeepxanax #xanaxsucks #ihatexanax #xanaxgirl #nothingbetterthanxanax
#buyxanaxonline #xanaxgo #mangiapregaxanax #wishihadxanax #xanaxman

#Liquidxanax #merryxanax #xanaxwillruinyourlifesostoptakingthemplease #wheresmyxanax! #xanaxnyc #antixanax #Myxanax #xanaxforsale #lixxanax #xanaxmariz #getoffxanax #xanaxanybody #instaxanaxdosis #xanaxmarket #buyxanax #needxanaxnow #passthedogxanax #xanax) #enexanax #xanaxjewlery #mangiapregaexanax? #naturalxanax #xanaxbars2 #iwishyoulovedmelikeyoudoxanax #ineedxanax!!! #nomorexanax, #fuckxanax} #xanaxvscaffiene #havexanaxtoo #xanaxkills #xanaxforsalehmu #onlyxanax #fueledbyxanax #lilxanaxfan #xanaxforanxiety #xanaxbarsbelike #lifeofxanax, #xanaxsmoothie #xanaxx #sometimesweneedxanax #🖕🏽xanax #gotxanax? #xanaxfamilyekaraoke #yellowxanax #AZxanax #allthexanaxplease #fuckxanax #passthexanax! #meandxanax #thankgodforxanax #coffeexanax #xanaxplease #xanaxmug #greenxanax #continuedoingxanax #fuckxanax. #enExanax #xanaxart #antixanaxmovement #whyxanax #xanaxproblems #xanaxpower #lilxanax. #followforxanax #Antixanax #noxanaxrequired #chiamatemixanax #xanaxgel #needxanax #xxxanax #hydrocodones #xanaxbarsforsale #ineedaxanaxandahug #funwithxanax #xanaxispoison #xanaxmoment #fuckxanax... #xanaxparty #liquidxanaxtotherescue #xanaxwave #xanax. #Donttakexanax #stopglorifyingxanax #xanaxsociety #xanaxszív #xanaxisthebest #chemondosarebbesenzaxanax #xanaxsex #mensxanax #xanaxneeded #xanaxoxycodine #30mgoxycodone #mentesanaincorporexanax #xanaxfamy #catxanax. #xanaxs #xanaxgrp #Stopdoingxanax2017 #wheresmyxanax #papaconxanax #Roxycodone #radsejxanax💊 #footballxanax #imoutofxanax #fuckxanaxs #xanaxdiego #stoptakingxanax #naturesxanax #xanaxbarstho #xanaxsystem #xanaxlovers #stopdoingxanax #Nomorexanax #Stopdoingxanax2018 #ilovexanax #checkxanaxthebandonfacebook #fuqxanax #xanaxiamoci #xanaxgeneration #xanaxUK #noxanaxforyou #xanaxGOD #xanaxsefxaristw #xanaxismyfriend #barbiexanax, #xanaxmomentcall #xanaxgotmethough #xanaxbarshawty #altrochexanax #xanaxnation #Barbiexanax #mynaturalxanax #yellowxanaxforsale #xanaxislife #ripxanaxboy #buyxanaxuk #saynotoxanax #xanaxcharm #liquidxanaxroller #liquidxanaxblend #xanaxdeath #liquidxanax #oxycodonewithoutprescription #NOxanax #xanaxmademedoit #rickysmusicismynaturalxanax #teamxanax #xanaxkid #darkxanax #noxanaxneeded #thisiswhatxanaxlookslike #xanaxthedog, #getxanax #dogxanax? #xanaxoverdose #xanaxcocktail #jebacxanax #xanaxboy #babyxanax. #dontdoxanaxkids #xanaxforsealife #hydrocodoneacetaminophen #hydrocodoneforsale #xanaxvolgograd #stopdoingxanax2017 #xanaxcake, #xanaxmemes

#xanaxgetsyouthroughlife #xanaxdreams #_thatisanawfullotofxanax
#xanaxbarslovers #dontdoxanax2017 #stopdoingxanax2018 #keepdoingxanax2017
#xanaxTV #popaxanax #xanaxtherapy #mynaturalxanax. #xanaxisbad
#xanaxandpatron #xanaxfami #stopusingxanax #liquidxanax... #Kawaixanax
#barbiexanax... #princessxanax #stopxanax2k18butifyoudontwanttohitmeup
#keepcalmandtakeaxanax #xanaxaddict #xanaxtheband #1mgxanax #xanax".
#xanaxcanvas #xanaxfacetattoo #takeaxanax #Noxanax #xanaxcandothat
#xanaxisstupid #xanaxhulks #xanaxnecklace #xanaxeyes #xanax? #xanaxbar
#whereismyxanax #oxycodone30mg #mountainxanax #xanaxmedov__0557237217AZTRAZ
#familyxanax #xanaxtattoo #tookxanax #xanaxtrapnight #myxanax,
#xanaxandmusclerelaxers #xanaxintheveins #xanaxgod #naturalxanax,
#leavexanax #xanaxbarsandbongrips #xanaxsupplier.com #xanaxforever
#xanaxpillow #percocert #hydrocodone, #xanaxlove- #xanaxgang #allthexanax
#xanaxsnowboards #enxanax #xanaxkaraokekuantan #wheresthexanax
#barbiexanaxdocet #xanaxedit #lixanax #stillneedsomexanaxtho #xanaxgetmehigh
#xanaxband #stopusingxanax2017 #xanaxnightmares #i❤️xanax
#takeaxanaxcalmdown #megliodelloxanax #toobadxanaxtasteslikepoison
#ascoltandobarbiexanax #xanaxforeveryone #th_xanax #ihatexanax.
#timeforxanax #xanaxgrp] #dontdoxanax2018 #xanaxfamilY #letsgetoffxanax
#xanaxchile #imxanax #weleavexanaxin2017 #greenxanaxbars #xanaxfamily
#ineedaxanax #xanaxflavours #mangiapregaxanax. #2mgxanax
#stopabusingxanax2017 #stopxanax #flappyxanax #xanaxtasia
#cheloxanaxsiaconte #xanaxbehavior #xanaxhigh #xanaxbaby #hydrocodoneAPAP
#anemelialogwxanax #hydrocodoneaddiction #xanaxbarsgood #enexanaxbersani
#gummiexanax #stopdoingxanax2017 #нуженxanax #xanaxworld #bitchimoff12xanax
#xanaxlovestory #xanaxjokes #xanaxevents #xanaxpentrusuflet #xanaxmood
#didnttakethexanax #lilxanax #xanaxatthemet #xanaxgrp]□ #xanaxbarz
#xanaxbarsandhenny #xanaxcake #catsonxanax #fuckoffxanax #ilinoisxanaxfamily
#babyxanax #xanaxtrump #xanaxplug #thexanax #enexanaxnonsiconoscevano
#fuckxanax2018. #herbalxanax #fuchxanax #youapussyifyoudontxanax #xanaxlife
#Betterthanxanax #xanaxplease😳 #whyismyxanaxspeakingspanish #xanaxlilpeep
#xanaxsmoking #wheresthexanax. #xanaxhere #xanaxfamilykaraoke #xanaxyellow
#xanax1mg #xanaxisthenewcamomile #xanaxmafrenz #xanaxuk #Needaxanax
#xanaxsavedmylife #xanaxmedov__0557237217 #musicxanax #xanaxbarspartii
#sweetiegangxanaxovekralovstvi #fuckxanaxrestinpeep #popexanax
#xanaxbarsfordaze #nomorexanax #xanax2 #Roxanax #hydrocodone #fuckyouxanax

#leavexanaxin2017 #needxanax. #xanaxcommunityofinstagram #iusedtopopxanaxlikecandy #yayforxanax #morexanaxplease #xanaxing. #itsactuallyjusthumanxanax #xanaxqueen #xanaxb #xanaxpot #dqxanaxtasia #xanaxa #xanaxlil #xanaxking #Fuckfakexanax #fuckxanaxlifetime #santaxanaxdelima #xanaxruinslives #xanaxbandung #xanaxwlean #xanax #ineedxanax #xanaxinspired #xanaxlover #actavisxanax #xanaxcantfixthis #halfnegenxanax #xanaxtv #streetxanax #flushurxanaxdownthetoilet #xanaxgraffiti #xanaxbitch #xanaxlondon #fuckxanax2017 #noxanax #noxanax, #hydrocodoneisnojoke #xanaxforlife #xanaxjazz #stopxanax2017 #peepxanax #fuckxanaxcrackheads #xanaxwithdrawals #instaxanax #Rarexanax #xanaxmusik #xanaxkuantan #herbalxanax, #xanax! #liquidxanax!!! #xanax, #xanaxlattetogo #xanax2mgbars #xanaxday #xanaxtree #telealsaporedixanaxoquasi #jualxanaxonline #bringmexanax #tattooxanax #gotxanax #oxycodone, #liquidxanax, #xanaxisadrug #xanaxloveya #dontdoxanax #fuckingxanax #xanaxalert #xanaxfootballs #thatsanawfullotofxanax #citazionibarbiexanax #xanaxepidemic #bluexanax #fuccxanax #naturesxanax. #oxycodone #xanaxfordepression #lovexanax #xanaxe #xanaxjob #labelxanax #xanaxbarselfie #xanaxbarsandalcohol #xanax0.25 #xanaxtasiagonorriah #xanaxbars2mg #donthaveanyxanax #xanaxkaraoke #xanaxgangs #livinlavidaxanax #xanaxandchill #tohighonxanax #barbiexanax! #antixanax? #xanaxtreatment #whitexanax #barbiexanaxassaggia #xanaxprincess #xanaxandlean #xanaxbabee🤔 #fuckxxanax #halloweenismyxanax #hydrocodone10mg #xanax+market=forevertogether #sundayisxanaxandchillday #xanaxfamilys #xanaxace #xanaxweedandalcohol #xanaxfamily. #xanaxliviuiancu #xanaxedout #xanaxiswaybetter #xanaxbomb #xanaxismybestfriend #mochavodkaxanaxlatte #xanaxfanleszekamigèlek #dogxanax #xanaxalldayeveryday #xanaxout #Enexanax #passthexanax #xanaxmusic #hydrocodonehigh #withoutthexanax #screwxanax #myxanax #fightoveraxanax #fuckxanax2018 #alberodixanax #вспоминаюxanax #xanaxdayout #FUCKxanax #xanaxfamilies #xanaxchy #noncirestachexanax #liquidxanaxessentialoil #thexanaxians #Lilxanax #doescostcosellxanax,getmesometoo #xanaxtorio #xanaxmusic. #xanaxietyedits #xanaxpills #jualxanax #xanaxisshit
